# Temporal trends in calls for suicide attempts to poison control centers in France during the COVID-19 pandemic: a nationwide study

**DOI:** 10.1007/s10654-022-00907-z

**Published:** 2022-08-30

**Authors:** Fabrice Jollant, Ingrid Blanc-Brisset, Morgane Cellier, Marine Ambar Akkaoui, Viet Chi Tran, Jean-François Hamel, Marie-Aude Piot, Mikail Nourredine, Patrick Nisse, The French Poison Center Control Research Group, Keith Hawton, Alexis Descatha, Dominique Vodovar

**Affiliations:** 1 Universitätsklinik für Psychiatrie und Psychotherapie, Universitätsklinikum Jena, Philosophenweg 3, 07743 Jena, Germany; 2 Nîmes Academic Hospital (CHU), Nîmes, France; 3 School of Medicine, Université Paris-Saclay, Le Kremlin-Bicêtre, France; 4 CHU Bicêtre, APHP, Le Kremlin-Bicêtre, France; 5 McGill Group for Suicide Studies, McGill University, Montréal, Canada; 6 Moods Team, INSERM UMR-1178, CESP, Le Kremlin-Bicêtre, France; 7 CHU Bordeaux, Aquitaine Poison Control Center, Bordeaux, France; 8 CHU Angers, Poison Control Center - Clinical Data Center, Angers, France; 9 UNIV Angers, CHU Angers, Univ Rennes, Inserm, EHESP, Irset (Institut de Recherche en Santé, Environnement et Travail)–UMR S1085 SFR ICAT, Angers, France; 10 Département de Psychiatrie et d′addictologie, AP-HP, GHU Paris Nord, Paris, France; 11 Laboratoire d’analyses et de Mathématiques Appliquées (LAMA), Gustave Eiffel University, Paris Est Creteil University, CNRS, Marne-la-Vallée, France; 12 Institut Mutualiste Montsouris & Université Paris Cité, Paris, France; 13 Epidemiological and Public Health Research Centre (CESP) - UMR 1018- UVSQ, Villejuif, France; 14 Biostatistics and Methodology Department, CHU Angers, Angers, France; 15 Service Hospitalo-Universitaire de Pharmacotoxicologie de Lyon, Hospices Civils de Lyon, Lyon, France; 16 Service de Recherche et Épidémiologie Clinique, Hospices Civils de Lyon, Lyon, France; 17 Laboratoire d’Évaluation et Modélisation des Effets Thérapeutiques, UMR CNRS 5558, Lyon, France; 18 CHU Lille, Poison Control Center, Lille, France; 19 Centre for Suicide Research, University of Oxford, Oxford, UK; 20 Paris Academic Hospital (APHP), Poison Control Center & Université Paris Cité, Paris, France; 21 UMRS-1144, Faculty of Medicine, Paris, France

**Keywords:** Attempted suicide, COVID-19, Poison Control Center, Surveillance, Age, Gender, Adolescent

## Abstract

Concerns have been raised about early vs. later impacts of the COVID-19 pandemic on suicidal behavior. However, data remain sparse to date. We investigated all calls for intentional drug or other toxic ingestions to the eight Poison Control Centers in France between 1st January 2018 and 31st May 2022. Data were extracted from the French National Database of Poisonings. Calls during the study period were analyzed using time trends and time series analyses with SARIMA models (based on the first two years). Breakpoints were determined using Chow test. These analyses were performed together with examination of age groups (≤ 11, 12–24, 25–64, ≥ 65 years) and gender effects when possible. Over the studied period, 66,589 calls for suicide attempts were received. Overall, there was a downward trend from 2018, which slowed down in October 2019 and was followed by an increase from November 2020. Number of calls observed during the COVID period were above what was expected. However, important differences were found according to age and gender. The increase in calls from mid-2020 was particularly observed in young females, while middle-aged adults showed a persisting decrease. An increase in older-aged people was observed from mid-2019 and persisted during the pandemic. The pandemic may therefore have exacerbated a pre-existing fragile situation in adolescents and old-aged people. This study emphasizes the rapidly evolving situation regarding suicidal behaviour during the pandemic, the possibility of age and gender differences in impact, and the value of having access to real-time information to monitor suicidal acts.

**Supplementary Information:**

The online version contains supplementary material available at 10.1007/s10654-022-00907-z.

## Introduction

The Coronavirus 2019 disease (COVID-19) broke out in China at the end of 2019 and rapidly became a pandemic. By the end of May 2022, more than 6.3 million people had died from COVID-19 and more than 530 million had been infected worldwide (https://ourworldindata.org/grapher/cumulative-covid-deaths-region).

COVID-19 has generated a lot of anxiety, depression and traumatic symptoms in the general population [1–6]. In order to limit contaminations, home confinement and social contact restrictions have been implemented in numerous countries, leading many people to experience social isolation. In addition, many individuals found themselves in financial difficulties, with consequent deleterious mental health effects [7]. In this context, understandable concerns have been voiced that the pandemic and associated consequences may lead to increasing numbers of suicidal acts [8–10]. Yet, initial data showed decreasing or stable rates of both suicide deaths [11] and non-fatal self-harming acts [12–14] during the early months of the pandemic in 2020. However, data from a few places across the world have shown a more recent increase in self-harm or suicide attempts in young people, notably female adolescents, since 2021 [15–19].

In France, the first official COVID-19 case was declared on January 24^th^, 2020. It led to a first strict lockdown from mid-March to mid-May 2020, which was followed by a decrease in the number of deaths and infected cases. Between May 2020 and December 2021, a series of additional lockdowns and night curfews, social restrictions and regulations, including requirement for a vaccination pass, regular closures of schools and universities, and compulsory working from home were implemented to contain four subsequent infectious waves. Data on self-harm hospitalizations in France between January and August 2020 showed an overall 8.5% decrease as compared to the same period in 2019, starting in the first week of the first lockdown [20]. However, it is still unclear if this decrease was actually related to the COVID-19 pandemic or represents the continuation of a prior decade-long decrease in self-harm in France [21]. Moreover, it has been shown that up to 40% of people do not present to hospital following a suicide attempt [22]. Therefore, a reduction in self-harm hospitalization does not preclude the possibility of increased suicidal acts. Following this early period, warning signs have been reported by clinicians about a possible increase in the number of mental health consultations among children [23]. Self-harm hospitalizations for the period September 2020-August 2021 showed a persistent decrease in middle-aged people but an important increase in female adolescents from January 2021 [19]. The prolonged effects of COVID-19 on suicide attempts need to be further examined.

One additional issue highlighted by the current COVID-19 pandemic is the lack of real-time data to rapidly monitor its impact on self-harming behaviors and guide healthcare responses [24], an issue not specific to France. It is of major importance to have access to rapid information systems in times of crises, whether epidemic, economic, environmental, or due to other causes. In France, availability of data on causes of deaths is delayed by four years, and by a few months for data on self-harm hospitalizations [25]. Calls for suicidal ingestions to Poison Control Centers (PCC) may be an interesting real-time monitoring system for suicide attempts. While the absolute numbers of calls do not represent an exact measure of the extent of suicidal behavior – only drug and other toxic overdoses are identified, and many suicidal ingestions do not result in phone calls to PCCs – monitoring trends over years may yield rapid warning signals. Of note, ingestions are involved in approximately 80% of all suicide attempts presenting to hospitals [26]. To our knowledge, only one study has used PCC data to assess the impact of the pandemic, this showing decreased numbers of calls in California in 2020 compared to 2019 and 2018 [27].

Our first objective was to assess trends in calls for suicide attempts during the COVID-19 pandemic (until May 31^st^, 2022) as compared to pre-pandemic years, using national data on calls to PCCs with regard to intentional ingestion of drugs or other toxic substances. We also aimed to investigate the relevance of using calls to PCCs as a means of monitoring suicide attempts at the national level [27]. Based on available data, we hypothesized an initial decrease in calls to PCCs mirroring data on self-harm hospitalizations, followed by a rebound in young people during the subsequent stage of the pandemic.

## Methods

### Data source

In France, eight PCCs respond to all calls from the public, caregivers and health authorities through a phoneline 24/7 about any type of toxic exposures. For each case, information is collected about the individual’s (‘henceforth referred to as ‘patient’) characteristics (age, gender) and exposure circumstances (i.e., accidental, recreational or suicidal). Data are then immediately stored in the French National Database of Poisonings (FNDP) administered by the French Ministry of Health. Cases are anonymously registered in the FNDP. Informed consent of the patients to collect their data and use them for research is waived in agreement with French law. The FNDP is registered and approved by the French ethic commission on data storage (*Commission Nationale Informatique et Libertés, CNIL*).

### Selection of cases

We extracted data from the FNDP on all cases of suicide attempts reported to the French PCCs from January 1^st^, 2018 to May 31^st^, 2022.

### Data collection

For each case of suicide attempt, we extracted the date of the suicide attempt, age (grouped as ≤ 11 / 12–24 / 25–64 / ≥ 65 years), and gender.

### Outcomes

The numbers of calls for suicide attempts each day were analyzed for the whole study period.

### Statistical Analyses

Statistical analyses were performed with R Studio® version 1.3.1093 for Windows® (version R 4.0.3). The number of monthly calls was considered as a time series with the “ts” function of the “stats” package in the R software®. All tests were two-sided, with a type I error threshold set at 0.05. Since potential differences according to gender and age groups have been shown in other studies, all analyzes were first performed on the whole sample then on the following subgroups: ≤ 11-year-olds, both genders (due to the small number of calls); males and females 12–24 years; males and females 25–64 years; > 65-year-olds, both genders (due to the small number of calls).

First, the “decompose” function of the “stats” package was used to decompose the trends of time series. After removing the trend of time series, we used Seasonal Autoregressive Integrated Moving Average (SARIMA). The ARIMA model assumes that once the series has been made stationary by removal of the trend and differentiation to suppress its integrated part, it is a linear combination of lags of observations, AR (autoregression), and lags of error, MA (moving average). SARIMA models are a seasonal variation of ARIMA accounting for the seasonality. We fitted SARIMA models on pre-pandemic data in France (1st January 2018 to 31st January 2020). The trend was estimated by a non-parametric moving average smoothing and an affine function was fitted. No differentiation of our series was needed. We selected the number of AR and MA lags to use in each SARIMA model via autocorrelation function (ACF), with the “ggAcf” function of the “forecast” package and partial autocorrelation function (PACF) plots, with the “ggPacf” function, respectively. For the chosen SARIMA model, we used the "auto.arima" function of the “forecast” package, and checked the normality of the residuals. Ljung-Box tests of autocorrelation of the residuals were run with the “checkresiduals” function, on each SARIMA model to assess goodness-of-fit. Predictions were then made, from the period prior to the COVID-19, to see how calls would have changed if the COVID 19 pandemic had not occurred. For this, we added to the forecasts of the SARIMA model the expected trend.

From the trend computed with the “decompose” function, one can see two affine behaviors at the beginning and end of the observation period. An affine function was fitted to the trend on these periods using least squares. Then, we performed Chow test with the “Fstats” function of the “strucchange” package to estimate periods of trend reversal, if any, using both forward and backward procedures where the breakpoint is determined starting from the first and last temporal observations, respectively. Final regression of the trend data was made before and after each identified breakpoint. Regression line coefficients were compared using the Fisher test, after having checked the normality of the residuals. The null hypothesis chosen was that there would be no difference between the whole sample and the subgroups.

## Results

### *Characteristics of the patients* (Table 1)

**Table 1:** Numbers of calls for suicide attempts to French Poison Centers from 1st January 2018 to 31st May 2022, per year and month, and by age group and gender. NA: No data available

	Total								2018							
Month	Number of calls for suicide attempt	Gender		Age groups					Number of calls for suicide attempt	Gender		Age groups				
		Female (N, %)	NA N (%)	≤ 11 N (%)	12–24 N (%)	25–64 N (%)	≥ 65 N (%)	NA N (%)		Female N %)	NA N (%)	≤ 11 N (%)	12–24 N (%)	25–64 N (%)	≥ 65 N (%)	NA N (%)
January	5665	3873 (68)	2 (0)	55 (1)	2912 (51)	2287 (40)	343 (6)	68 (1)	1492	989 (66)	2 (0)	8 (1)	696 (47)	685 (46)	92 (6)	11 (1)
February	4791	3272 (68)	3 (0)	47 (1)	2459 (51)	1939 (40)	289 (6)	57 (1)	1158	744 (64)	0 (0)	8 (1)	515 (44)	551 (48)	71 (6)	13 (1)
March	5577	3818 (68)	6 (0)	73 (1)	2988 (54)	2132 (38)	341 (6)	43 (1)	1529	1025 (67)	1 (0)	18 (1)	719 (47)	688 (45)	96 (6)	8 (1)
April	4746	3254 (69)	2 (0)	67 (1)	2332 (49)	1958 (41)	343 (7)	46 (1)	1348	904 (67)	1 (0)	20 (1)	574 (43)	645 (48)	100 (7)	9 (1)
May	5082	3593 (71)	1 (0)	75 (1)	2709 (53)	1901 (37)	340 (7)	57 (1)	1392	976 (70)	1 (0)	20 (1)	681 (49)	585 (42)	87 (6)	19 (1)
June	4929	3421 (69)	6 (0)	73 (1)	2542 (52)	1966 (40)	293 (6)	55 (1)	1378	929 (67)	1 (0)	17 (1)	656 (48)	626 (45)	71 (5)	8 (1)
July	4065	2718 (67)	3 (0)	43 (1)	1798 (44)	1868 (46)	315 (8)	41 (1)	1053	701 (67)	0 (0)	10 (1)	408 (39)	545 (52)	83 (8)	7 (1)
August	4174	2792 (67)	0 (0)	32 (1)	1868 (45)	1890 (45)	333 (8)	51 (1)	1171	782 (67)	0 (0)	6 (1)	462 (39)	588 (50)	100 (9)	15 (1)
September	4799	3281 (68)	3 (0)	47 (1)	2458 (51)	1924 (40)	310 (6)	60 (1)	1286	842 (65)	0 (0)	9 (1)	568 (44)	613 (48)	87 (7)	9 (1)
October	4734	3244 (69)	3 (0)	37 (1)	2338 (49)	1967 (42)	323 (7)	69 (1)	1250	828 (66)	0 (0)	3 (0)	523 (42)	625 (50)	88 (7)	11 (1)
November	5112	3671 (72)	8 (0)	62 (1)	2964 (58)	1735 (34)	299 (6)	52 (1)	1161	811 (70)	3 (0)	16 (1)	619 (53)	452 (39)	67 (6)	7 (1)
December	4512	3089 (68)	5 (0)	39 (1)	2357 (52)	1741 (39)	325 (7)	50 (1)	1043	691 (66)	0 (0)	11 (1)	497 (48)	464 (44)	64 (6)	7 (1)
Total	58,186	40,026 (69)	42 (0)	650 (1)	29,725 (51)	23,308 (40)	3854 (7)	649 (1)	15,261	10,222 (67)	9 (0)	146 (1)	6918 (45)	7067 (46)	1006 (7)	124 (1)

	**2019**								**2020**							
Month	Number of calls for suicide attempt	Gender		Age groups					Number of calls for suicide attempt	Gender		Age groups				
		Female (N, %)	NA N (%)	≤ 11 N (%)	12–24 N (%)	25–64 N (%)	≥ 65 N (%)	NA N (%)		Female N %)	NA N (%)	≤ 11 N (%)	12–24 N (%)	25–64 N (%)	≥ 65 N (%)	NA N (%)
January	1469	984 (67)	0 (0)	15 (1)	710 (48)	629 (43)	99 (7)	16 (1)	1346	940 (70)	0 (0)	13 (1)	719 (53)	510 (38)	80 (6)	24 (2)
February	1160	770 (66)	0 (0)	19 (2)	555 (48)	524 (45)	52 (4)	10 (1)	1120	756 (68)	3 (0)	9 (1)	573 (51)	435 (39)	88 (8)	15 (1)
March	1372	924 (67)	1 (0)	15 (1)	724 (53)	546 (40)	78 (6)	9 (1)	1132	763 (67)	1 (0)	12 (1)	580 (51)	436 (39)	86 (8)	18 (2)
April	1119	777 (69)	0 (0)	18 (2)	586 (52)	438 (39)	70 (6)	7 (1)	966	628 (65)	1 (0)	9 (1)	418 (43)	437 (45)	86 (9)	16 (2)
May	1138	808 (71)	0 (0)	18 (2)	605 (53)	439 (39)	71 (6)	5 (0)	1020	670 (66)	0 (0)	9 (1)	452 (44)	442 (43)	106 (10)	11 (1)
June	1122	772 (69)	1 (0)	14 (1)	523 (47)	500 (45)	69 (6)	16 (1)	1089	752 (69)	1 (0)	14 (1)	530 (49)	455 (42)	80 (7)	10 (1)
July	981	647 (66)	1 (0)	8 (1)	418 (43)	468 (48)	69 (7)	18 (2)	868	577 (66)	0 (0)	12 (1)	387 (45)	387 (45)	75 (9)	7 (1)
August	917	581 (63)	0 (0)	10 (1)	379 (41)	442 (48)	76 (8)	10 (1)	937	646 (69)	0 (0)	5 (1)	430 (46)	423 (45)	69 (7)	10 (1)
September	1055	736 (70)	1 (0)	11 (1)	522 (49)	440 (42)	68 (6)	14 (1)	1112	727 (65)	2 (0)	12 (1)	526 (47)	470 (42)	80 (7)	24 (2)
October	1138	787 (69)	1 (0)	14 (1)	541 (48)	483 (42)	72 (6)	28 (2)	1081	711 (66)	2 (0)	11 (1)	524 (48)	456 (42)	76 (7)	14 (1)
November	1253	902 (72)	3 (0)	15 (1)	716 (57)	427 (34)	78 (6)	17 (1)	1234	855 (69)	0 (0)	15 (1)	704 (57)	439 (36)	67 (5)	9 (1)
December	1084	724 (67)	3 (0)	7 (1)	530 (49)	440 (41)	76 (7)	31 (3)	1110	775 (70)	1 (0)	10 (1)	593 (53)	413 (37)	82 (7)	12 (1)
Total	13,808	9412 (68)	11 (0)	164 (1)	6809 (49)	5776 (42)	878 (6)	181 (1)	13,015	8800 (68)	11 (0)	131 (1)	6436 (49)	5303 (41)	975 (7)	170 (1)

	2021								2022							
Month	Number of calls for suicide attempt	Gender		Age groups					Number of calls for suicide attempt	Gender		Age groups				
		Female (N, %)	NA N (%)	≤ 11 N (%)	12–24 N (%)	25–64 N (%)	≥ 65 N (%)	NA N (%)		Female N %)	NA N (%)	≤ 11 N (%)	12–24 N (%)	25–64 N (%)	≥ 65 N (%)	NA N (%)
January	1358	960 (71)	0 (0)	19 (1)	787 (58)	463 (34)	72 (5)	17 (1)	1796	1369 (76)	2 (0)	17 (1)	1224 (68)	444 (25)	92 (5)	19 (1)
February	1353	1002 (74)	0 (0)	11 (1)	816 (60)	429 (32)	78 (6)	19 (1)	1466	1077 (73)	0 (0)	10 (1)	944 (64)	414 (28)	89 (6)	9 (1)
March	1544	1106 (72)	3 (0)	28 (2)	965 (63)	462 (30)	81 (5)	8 (1)	1746	1296 (74)	2 (0)	17 (1)	1158 (66)	485 (28)	73 (4)	13 (1)
April	1313	945 (72)	0 (0)	20 (2)	754 (57)	438 (33)	87 (7)	14 (1)	1479	1093 (74)	0 (0)	18 (1)	896 (61)	481 (33)	75 (5)	9 (1)
May	1532	1139 (74)	0 (0)	28 (2)	971 (63)	435 (28)	76 (5)	22 (1)	1833	1424 (78)	0 (0)	16 (1)	1207 (66)	489 (27)	104 (6)	17 (1)
June	1340	968 (72)	3 (0)	28 (2)	833 (62)	385 (29)	73 (5)	21 (2)								
July	1163	793 (68)	2 (0)	13 (1)	585 (50)	468 (40)	88 (8)	9 (1)								
August	1149	783 (68)	0 (0)	11 (1)	597 (52)	437 (38)	88 (8)	16 (1)								
September	1346	976 (73)	0 (0)	15 (1)	842 (63)	401 (30)	75 (6)	13 (1)								
October	1265	918 (73)	0 (0)	9 (1)	750 (59)	403 (32)	87 (7)	16 (1)								
November	1464	1103 (75)	2 (0)	16 (1)	925 (63)	417 (28)	87 (6)	19 (1)								
December	1358	942 (69)	1 (0)	16 (1)	765 (56)	472 (35)	93 (7)	12 (1)								
Total	16,185	11,635 (72)	11 (0)	214 (1)	9590 (59)	5210 (32)	985 (6)	186 (1)	8320	6259 (75)	4 (0)	78 (1)	5429 (65)	2313 (28)	433 (5)	67 (1)

Between January 1st, 2018 and May 31st, 2022, 844,902 cases of toxic exposure were reported to the eight French PCCs, including 66,589 (7.9%) for suicide attempts (N = 15,261 in 2018, 13,808 in 2019, 13,015 in 2020, 16,185 in 2021 and 8,320 between January 1st and May 31st, 2022). Of the calls for attempted suicide, 77.2% (N = 51,408) were from health professionals and 22.8% (N = 15,181) from the public.

Suicide attempts involved women in 69.6% of cases (N = 46,328). The distribution of patients by age groups was: 1.1% aged ≤ 11 years, 52.8% 12–24 years, 38.6% 25–64 years and 6.4% 65 years and over (unknown age in 1.1% of cases).

The median number of substances involved in the suicide attempts was 2 (interquartile range [1;3]). At least one substance was a pharmaceutical drug in 82.2% of cases (N = 54,703), ethanol in 10.7% (N = 7145), and home cleaning products (including disinfectants) in 7.8% (N = 5193). Suicide attempts involving alcohol-based hand sanitizers were rare, but more than doubled in 2020 (N = 129) and 2021 (N = 155) compared to 2018 and 2019 (N = 55 and 63, respectively).

The average number of daily calls over the four-year period was 41.3.

### Time trends and time series modeling in the whole population

**Fig. 1:**
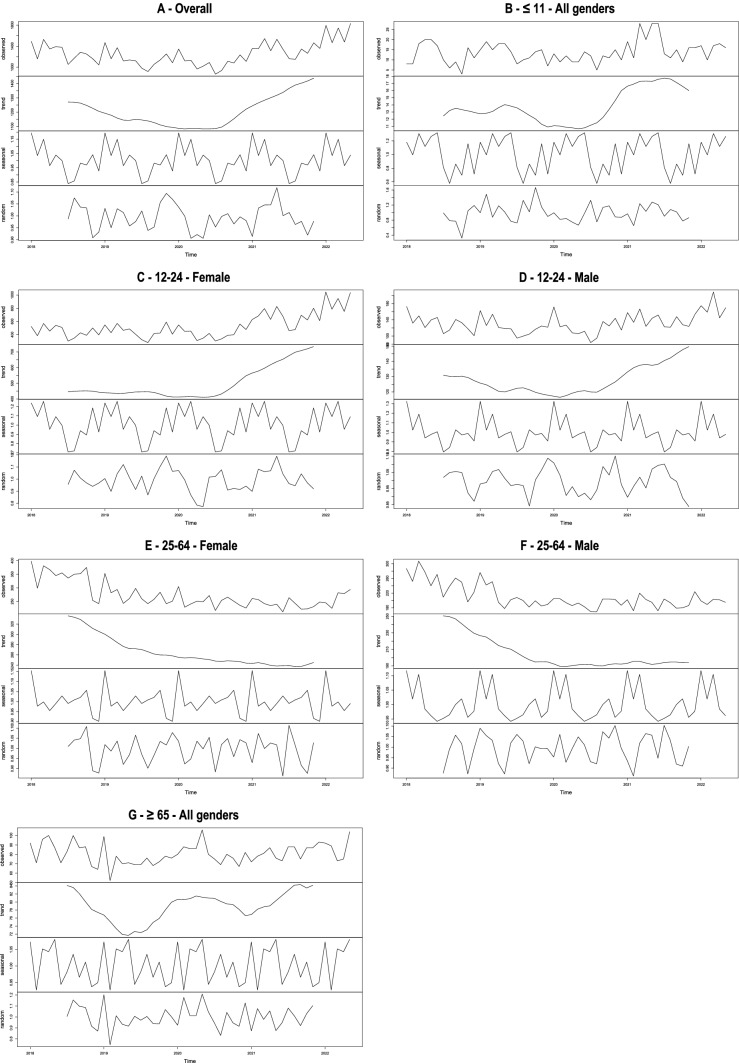
Decomposition of multiplicative time series for the whole sample (**A**), and by age and gender (**B–G**)

**Fig. 2:**
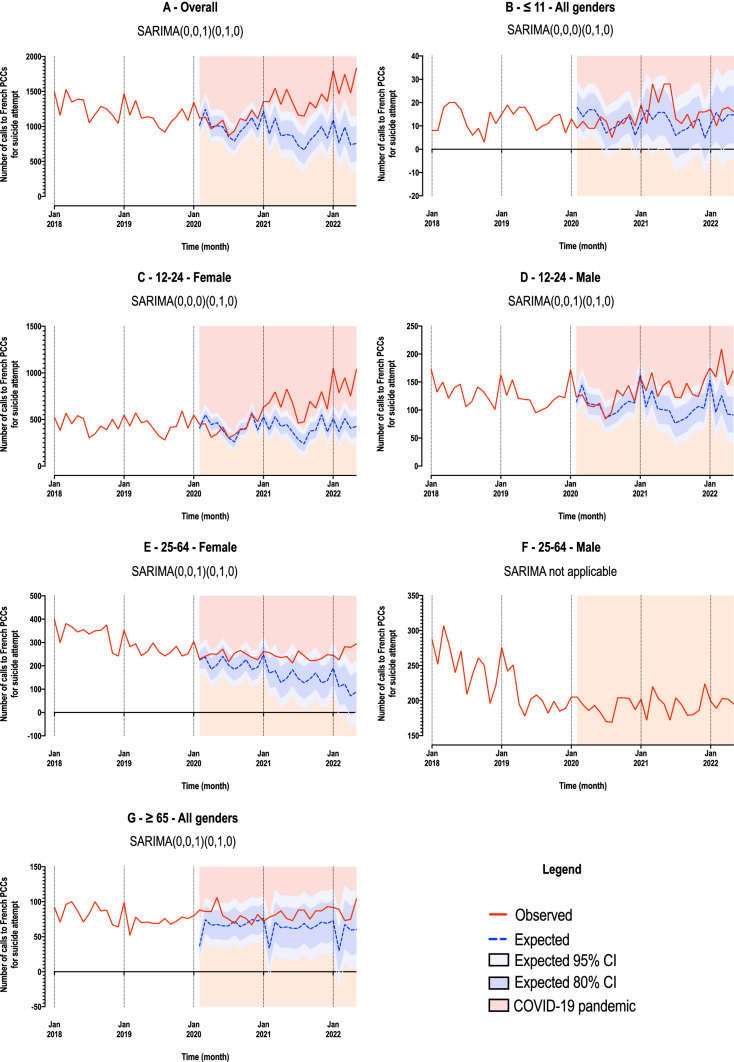
Time series modeling based on SARIMA prediction, in the whole sample (**A**) and by age and gender (**B–G**)

**Fig. 3:**
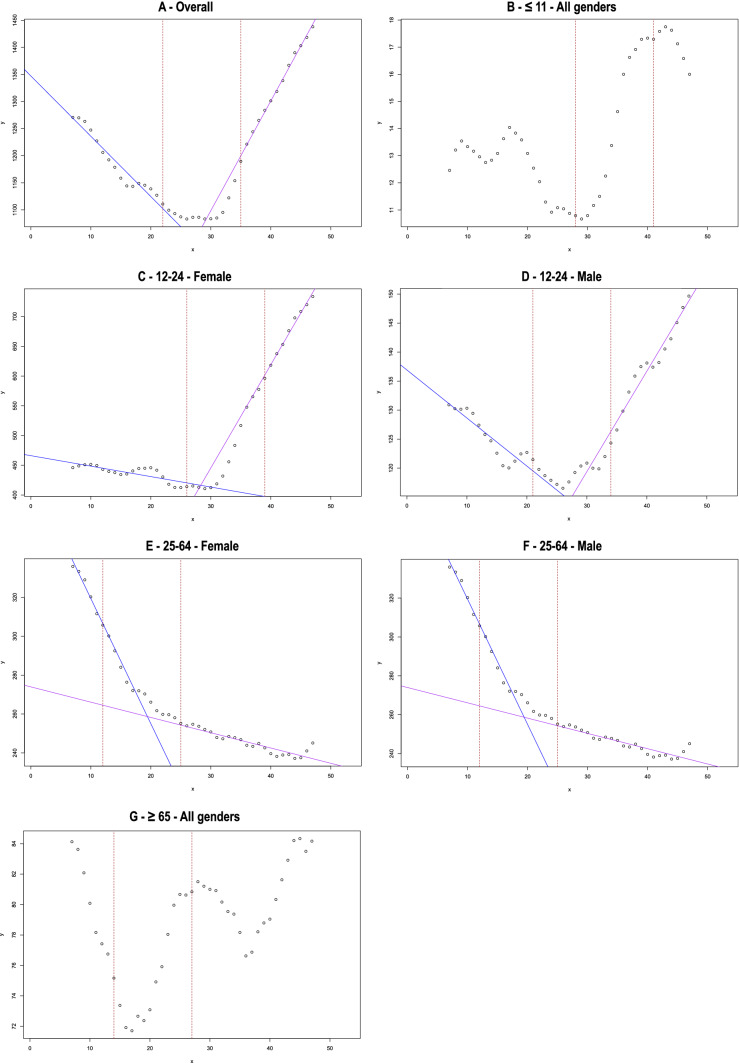
Illustration of Chow test using forward and backward linear regressions, in the whole sample (**A**) and by age and gender (**B–G**)

From the time series decomposition, a general trend emerged. The number of calls decreased then slowed down, then increased (Fig. 1A). The forecast based on SARIMA model was different from the actual observations, with higher number of calls than expected during the COVID period (Fig. 2A). The Chow test determined 2 breakpoints (Fig. 3A and Table 2), in October 2019 and November 2020. The coefficients of the decreasing and increasing slopes were both significant (Table 2) (see supplemental material for autocorrelation analyses).

**Table 2:** Chow test using forward (Chow test 1) and backward procedures (Chow test 2). Linear regression was performed from the first temporal period to the first breakpoint (Regression line 1) and from the second breakpoint to the last temporal observation (Regression line 2)

	Chow test 1 (forward procedure)	Chow test 2 (backward procedure)	Regression line 1 (from the first temporal observation to the first breakpoint obtained by forward procedure)			Regression line 2 (from the second breakpoint to the last temporal observation obtained by backward procedure)		
			Ordinate at origine	Slope	Different from 0 *p*-value	Ordinate at origine	Slope	Different from 0 *p*-value
Overall	October 2019	November 2020	1347.28	– 11.14	***	490.53	20.27	***
≤ 11	NA	NA	NA	NA	NA	NA	NA	NA
12–24—Female	February 2020	March 2021	466.18	– 1.760	***	– 75.43	17.36	***
12–24—Male	September 2019	October 2020	136.93	– 0.83	***	67.46	1.73	***
25–64—Female	December 2018	January 2020	383.84	– 6.44	***	273.97	– 0.79	***
25–64—Male	April 2019	May 2020	281.69	– 4.16	***	185.40	0.20	**
≥ 65	NA	NA	NA	NA	NA	NA	NA	NA

NA: Too few patients in the subgroup to perform the Chow test, *p* < 0.05; **: *p* < 0.01; ***: *p* < 0.001

### Analyses by age and gender

Patterns of time trends, forecasts and breakpoints differed according to age and gender.

In individuals aged 11 years and below, a decrease was found until the end of 2019 followed by an increase from mid-2020 (Fig. 1B). However, statistics could not be run in this age group due to the small number of calls (Table 2). The predicted trends did not differ from observations during the COVID period (Fig. 2B) and no break point could be identified (Fig. 3B).

Among 12–24-year-olds, a decrease followed by an increase in calls was observed (Fig. 1C-D). Decreases and increases were significant in both males and females (Table 2). However, the increase in females was particularly marked and explained most of the increase in calls observed during the COVID period (Fisher test, *p* = 0.855) (Table 2). Observations were higher than expected during the COVID period, notably in females (Fig. 2C-D). Breakpoints differed according to gender, with earlier time points in males: February 2020 and March 2021 in females, September 2019 and October 2020 in males (Fig. 3C-D and Table 2).

Trends were different in 25–64-year-olds from other age groups. There was a decrease in calls in females during the whole period and a decrease followed by a levelling off in males (Fig. 1E-F). All slopes were significant (Table 2). The numbers of calls in females remained within the confidence interval (Fig. 2E; statistical analysis not feasible in males). Two different break points were found in females and males: in December 2018 and January 2020 and in April 2019 and May 2020, respectively (Fig. 3E-F and Table 2).

Finally, trends were complex in people aged above 65-year-old. Following an initial decrease until mid-2019, an increase was observed that peaked in mid-2020, followed by a small decrease, and then another increase from early 2021 (Fig. 1G). However, statistical analyses could not be conducted in this age group due to the small number of calls (Table 2). The overall number of calls remained within the confidence interval (Fig. 2G) and no breakpoint could be identified (Fig. 3G).

## Discussion

Our study—which includes a lengthy overall period from 1^st^ January 2018 to 31^st^ May 2022, a two-year pre-pandemic period and more than two years of the COVID pandemic following the first official cases in February 2020—has highlighted several important findings concerning calls to PCCs in France for suicide attempts. Overall, there was initially a decrease in number of calls to PCCs, mirroring the reported reduction in hospitalizations for self-harm over the last ten years in France [21]. This decrease started to slow down around October 2019 but continued during the first months of the pandemic. A previous study showed a significant reduction in hospitalizations for self-harm in France during the period January to August 2020 (early months of the pandemic and first confinement) as compared to the same period in 2019, with a more marked reduction in women and a similar pattern in all age groups except older people [20]. This is also in keeping with several reports from other countries regarding hospitalizations or Emergency Room visits [12–14]. Then, a substantial increase was observed from November 2020 that continued until the end of the study period more than 18 months later. Again, this increase is very similar to which was observed for self-harm hospitalizations [19]. Therefore, two phases can be identified during the pandemic: a decrease during the first months continuing historical trends, and an increase since the end on 2020. The overall outcome is an excess number of calls for suicide attempts during the COVID-19 pandemic as compared to what was expected.

A second important finding is that this U-shaped curve in calls to PCCs was very different according to age and gender. Notably, the major increase in calls during the second part of the pandemic was largely related to calls for 12–24-year-old females. This explained most of the observed increase in calls. Similar results have been found in Japan for suicide deaths: following an initial decrease in the number of suicides (– 14%), there was a subsequent increase which started during the second COVID-19 wave in females (+ 37%) and in children/adolescents (+ 49%) during school closures [28]. Recent studies of trends in self-harm or suicide attempts have also found significant increases in female adolescents a few months after the start of the pandemic [15–18]. One possible explanation may be that physical distancing and restrictive measures, including closure of schools and universities and change in education over long periods, and the perspective that the pandemic would last much longer than expected, may have had a major impact in an age group for which social interactions are particularly important. In Chinese college students, an increase in depressive symptoms found after as compared to before the onset of COVID-19 was associated with boredom and emotional loneliness, but not lockdown per se [29]. In 12–24-year-olds in Switzerland, perceived stress during the first months of the COVID-19 pandemic was related to disruption of social life and activities [30]. In a qualitative study conducted in English adolescents during the first months of the pandemic, disruption and changes to school and education emerged as a major theme of concerns recorded in diaries [31]. These factors may have exacerbated the risk of self-harm in many individuals, as isolation and loneliness were identified as one of the main factors contributing to self-harm during the COVID-19 pandemic [32].

In contrast, calls related to suicide attempts in middle-aged males and females continued to decrease during the second phase of the pandemic, although at a slower rate than during the initial phase. One explanation may be that the important economic effort to support employment may have buffered the negative impact of COVID-19 in this age group. Future analyses should examine the impact of social and financial measures on self-harm during the COVID-19 pandemic.

Interpretation of trends in older-aged people is more complicated due to the small number of calls. However, this group was showing a significant increase as early as mid-2019 which was then amplified and persisted during the period of the COVID-19 pandemic that we have investigated. This population, which was at major risk of mortality due to COVID-19, may have particularly suffered from the isolation related to social restrictions. It will be important to see if data on suicides, when available, is consistent with these negative outcomes.

Lastly, it was surprising to find out that a first inflexion in the initial declining curves in numbers of call to PCCs was observed in all groups and both genders *before* the official start of the COVID-19 pandemic: in February 2020 in 12–24-year-old females but September 2019 in 12–24-year-old males, December 2018 in 25–64-year-old females, and April 2019 in 25–64-year-old males. These findings suggest that the negative impact of COVID-19, notably in adolescents and older people, may actually have occurred against a pre-existing fragile situation when the historical decline in self-harming behaviors was slowing down. It is hoped that the national suicide prevention strategy introduced in 2018 in France (https://solidarites-sante.gouv.fr/prevention-en-sante/sante-mentale/la-prevention-du-suicide/article/la-politique-de-prevention-du-suicide) will contribute to a reduction in suicide attempts in the next months.

Strength of this study includes the complete and national nature of the database, the rapid availability of data, and the interviews conducted by a specialist with the callers to determine the suicidal, recreational or accidental nature of the acts (as opposed to hospital data based on ICD-10 codes, for which the suicidal intention of self-harming behaviors is unknown). As mentioned above, a major limitation is that suicidal acts involving means other than overdose would not have been recorded. Moreover, not all suicidal ingestions give rise to a PCC call. Factors contributing to calls to PCCs from the public should be investigated. It is possible that these may have influenced findings in as yet unknown ways. Finally, the limited numbers of cases, especially in some age groups, necessitate cautious interpretation.

In conclusion, our analyses of calls to PCCs for a suicidal act highlights two different phases during the first two years of the COVID-19 pandemic, with differential age and gender effects. The early months of the pandemic were characterized by a significant decrease in calls, in line with reports from emergency room visits and hospitalization data, suggesting a true decrease in the number of suicidal acts that added to an already decreasing pattern in 2018 and 2019. One important exception was older adults, in whom a worrying increase was observed. Then, a second phase starting at the end of 2020 was characterized by a major increase in calls largely related to suicidal acts in young females, while calls for middle-aged people continued to decrease. Finally, it is possible that the negative effects of COVID-19 occurred in a pre-existing fragile situation in terms of suicide attempt prevention in adolescents and older people. This study emphasizes the complex and varying nature of the impact of a pandemic and related protective measures on suicidal acts. It also highlights the importance of real-time data to monitor suicidal acts—and the relevance of using calls to PCCs for this, especially when data need to be obtained rapidly.

## Supplementary Information

Below is the link to the electronic supplementary material.

Supplementary file1 (DOCX 163 kb)

## Data Availability

Anonymized data can be obtained on request to Dr Dominique VODOVAR after publication.
