# Supplementary figures and images for: Temporal trends in calls for suicide attempts to poison control centers in France during the COVID-19 pandemic: a nationwide study
Source: Eur J Epidemiol. 2022 Aug 30;37(9):901–13. doi: 10.1007/s10654-022-00907-z (PMC9425826; doi:10.1007/s10654-022-00907-z)

**Supplemental material**

Autocorrelation analyses for the whole sample and by age and gender


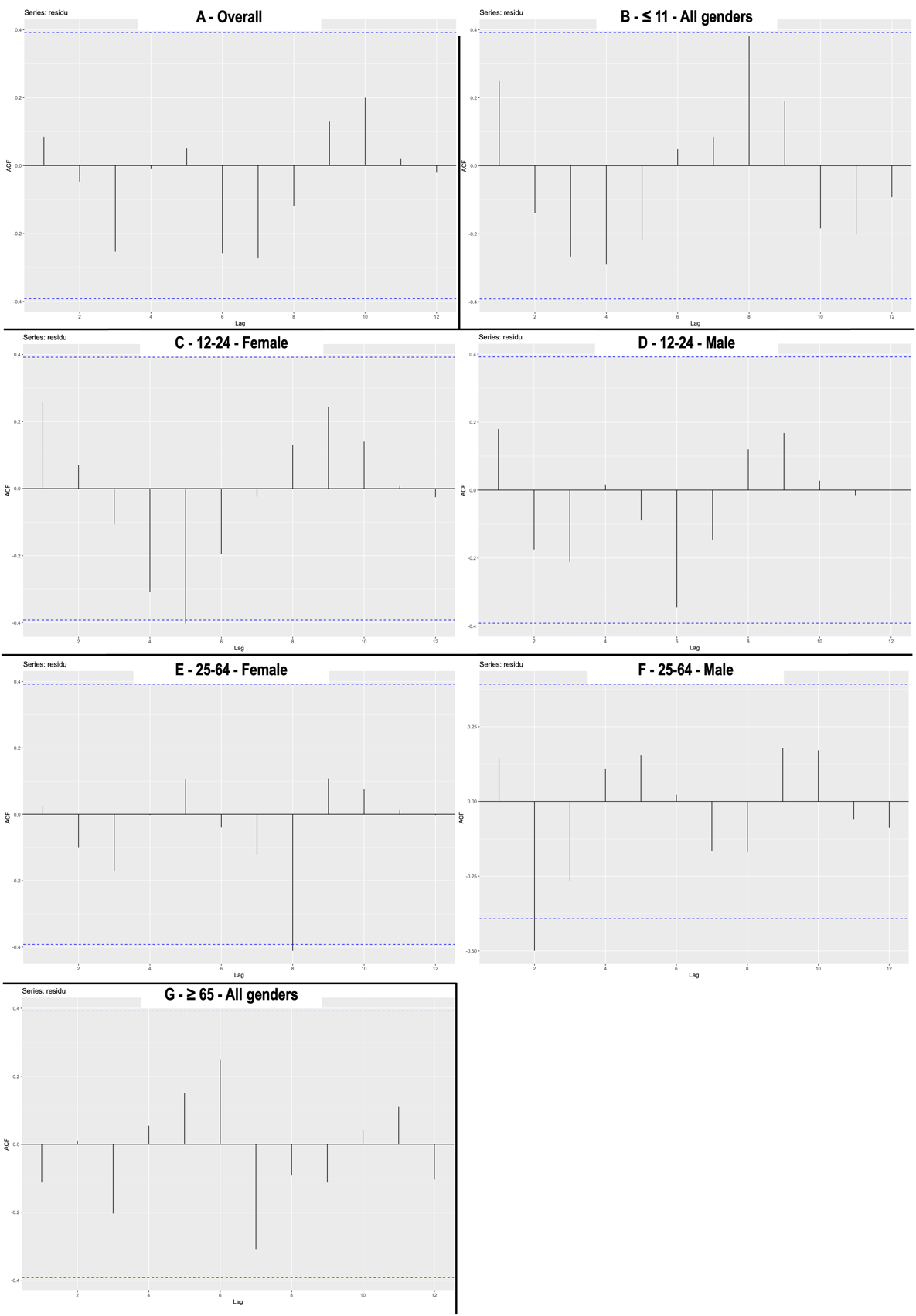

Supplement: Supplementary file 1 — Supplementary file1 (DOCX 163 kb) [file 10654_2022_907_MOESM1_ESM.docx]
